# How can a top-down government program can be sustainable: A case study of horticulture village program in South Sulawesi Province

**DOI:** 10.1371/journal.pone.0313993

**Published:** 2025-02-28

**Authors:** Valeriana Darwis, Chairul Muslim, Lyli Mufidah, Retna Qomariah, Yanti R. Darsani, Suharyon, Adri, Firdaus, Viktor Siagian, Juni Hestina, Saptana, Ashari, Muhammad Syakir

**Affiliations:** 1 Research Center for Behavioral and Circular Economics, National Research and Innovation Agency (BRIN), South Jakarta, Special Capital Region of Jakarta, Indonesia; 2 Research Center for Macroeconomics and Finance, National Research and Innovation Agency (BRIN), South Jakarta, Special Capital Region of Jakarta, Indonesia; 3 Research Center for Cooperatives, Corporations, and People’s Economics. National Research and Innovation Agency (BRIN), South Jakarta, Special Capital Region of Jakarta, Indonesia; 4 Research Center for Food Crops, National Research and Innovation Agency (BRIN), Cibinong, West Java, Indonesia; National Cheng Kung University, TAIWAN

## Abstract

The horticulture village program is one of the activities in increasing sustainable vegetable production. The activity was carried out in 2021 and one of the provinces that carried it out was the province of South Sulawesi. The purpose of the paper is to evaluate the sustainability of the horticulture village program from four dimensions, namely input, process, product, context/outcome, using the Rapfish analysis tool, and strengthened by partial budget analysis to see the magnitude of changes in revenue. From the results of the Rapfish analysis, it could be seen that horticultural village activities are less sustainable, with the lowest value in the product dimension. The main reason for this is that the planning and implementation of the program lacked a well-thought-out social process. It requires the need for improvement in terms of quantity and quality of seeds, which considers the suitability and habits of farmers. Assistance from extension workers related to the use of appropriate and periodic technology for plant conditions (product) which will have an impact on productivity and income (context) needs to be considered. The increase in income for chili and shallots due to following the recommendations of cultivation technology, including reducing the use of chemical fertilizers, increasing the use of organic fertilizers, reducing the use of chemical pesticides and using plastic mulch, can be an entry point to convince farmers of the sustainability of this program with further improvements.

## Introduction

Vegetable crops (*Olericulture*) are one of the horticultural subsector crop groups that are widely cultivated by the people of Indonesia. This plant also has potential competitiveness that is always able to achieve high competitiveness if the economy is stable without recession. Several studies showing that vegetable commodities have high competitiveness, both from the perspective of comparative and competitive advantages such as shallot [1], chili [2], potato [3]. This condition causes vegetable crops to be one of the subsectors that play an important role in supporting the country’s economy because it has high economic value and can be a source of income for the farming community. The importance of vegetable crops is also seen during the Covid-19 outbreak, where people consume a lot of vegetables in increasing the body’s immune system [4–7].

Vegetable plants have a source of vitamins and minerals that are very important to meet human nutritional intake. Therefore, its availability is increasing following the increase in population, income levels and people’s nutritional awareness. The availability of vegetables is greatly influenced by the production produced, number of vegetables exported and number of vegetables imported from outside the province. While vegetable production has had problems: farmers cultivate it on narrow land, limited capital, not yet maximized in overcoming pests, and low adoption of cultivation technology, especially in the use of production inputs and post-harvest handling that has not been maximized [8–10]. To maintain availability of vegetable production, programmatic, directed, integrated, and sustainable production activities are needed. The development of agriculture-based businesses is very important to be carried out in supporting the welfare of farmers [11].

The welfare of farmers is an economic aspect that supports the sustainability of vegetable cultivation in Indonesia. Additionally, social, institutional, and environmental aspects also need to be considered. One important factor is access to marketing or distribution channel of horticultural products, which represent social and institutional aspects, that can improve the performance of vegetable cultivation [12]. From an environmental perspective, wise use of agrochemicals is crucial. [13] highlights the substantial adverse impacts resulting from agrochemical use. Since vegetables typically use more agrochemicals than rice, the impacts are likely greater for vegetable cultivation. The results of the study by [14] indicate a shift from 1990 to2019 in the portion of agricultural value, moving from staple foods to high-value commodities, particularly vegetable.

One of the programs to increase sustainable vegetable production in one area implemented by the government is the horticulture village. The purpose of horticultural village development is stated in the context of evaluation/outcome, namely increasing productivity, product quality and income leading to the formation of a cooperative-based horticultural area (*one village one variety)*. This is expected to be a driver for the growth of new horticultural areas. In order to succeed, the commodities grown are in accordance with the agroecosystem and the habits of the community or farmers willing to plant and care for these commodities, as well as support from the local government.

The form of activity of the horticultural village program is the provision of assistance to farmers through farmer groups. The assistance includes the provision of seeds of superior varieties, production facilities, environmentally friendly plant disturbing organism control materials and assistance by extension workers. The horticulture village program is a *top-down program* that has been implemented since 2021. The top-bottom strategy, according to [15], is the concern of ruling groups to assimilate and integrate subordinate groups into the prevailing ideology in order to guarantee their own sustainability and security. The implementation of the top down program on agriculture has its own dilemma in Indonesia, which sometimes ignores social reconstruction [16, 17]. In order for the program to be sustainable, it is necessary to analyze of performance and prospects for sustainability in one of the provinces that have implemented the program [18, 19].

Numerous valuation systems and models have been employed to assess the initiatives, programs, or operations of institutions [20]. One of the approaches to assist managers in making informed judgments regarding the program has been introduced firstly by Stufflebeam [21]. The Context, Input, Process, and Product Evaluation Model (CIPP) is the name of his assessment approach. Such a model is supposed to be effectively used for evaluating the sustainability of the Horticulture Village program. Another paradigm that can be used is the stucture, conduct, performance (SCP) model [22], but this study focuses more on output and outcome indicators. Therefore, this paper aims to assess the horticultural village program’s sustainability from four angles: context/outcome, input, process, and product (CIPP) in South Sulawesi Province.

The novelty that is expected from this study is to provide input on variables that need to be prioritized from the top-down government’s programs that carried out outside Java to be sustainable. This is important because food centers in Indonesia are still focusing on Java. The use of partial budgeting in supporting Rapfish’s analysis is also a novelty itself, to ensure that the main stakeholders, namely farmers, do not suffer losses from the implementation of this program.

In detail, the objectives of this study include: (1) evaluate the performance of the success and sustainability of the implementation of the horticultural village development program; (2) Identify the problems faced, both technical and non-technical problems and leverage points for the sustainability of the horticultural village development program; and (3) Examining the impact of the implementation of the horticultural village development program on farmers’ income.

## Methodology

### Frame of thought

**Fig 1:**
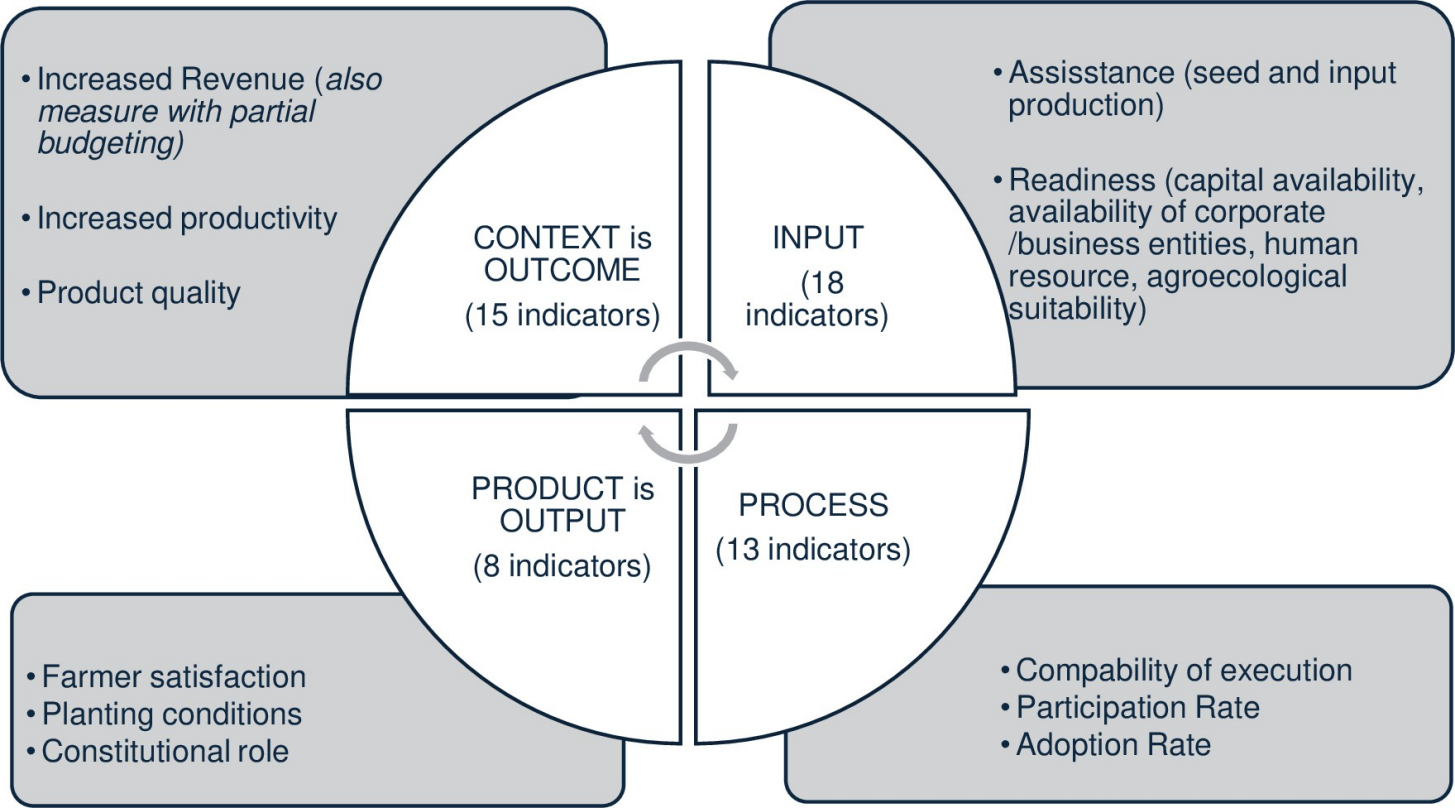
Frame of thought. Source: Adapted from [20] with variables adjusted to the composition of the study (author composition).

Evaluation activities should contain the principles of relevance, efficiency, effectiveness, results, impact and sustainability. The most important purpose of evaluation, is not to prove the right or wrong of the program but to improve the system. The focus of evaluation on each dimension namely input, process, product and context (which is the purpose /outcome) is carried out in order to evaluate the results of the program as a whole. The complete thinking line of evaluation of the horticultural village development program is presented in Fig 1 [23].

Literally, evaluation is a systematic assessment of an object. A program, on the other hand, is a planned mix of activities and resources intended to achieve specific objectives within a specified time and cost budget [24]. Thus, program/activity evaluation is the systematic assessment of a program/activity through the results of the implementation of the activities of each program. Evaluation of program implementation as a unitary tool to achieve goals in accordance with the object of evaluation and the purpose of carrying out implementation evaluation as [25] argues that the most important purpose of evaluation is not to prove the right or wrong of the program but to improve the system.

### Time, location, data and data source

The study was conducted from September to December 2022 in South Sulawesi Province. The determination of the location of the study was carried out *purposively* with the criteria that South Sulawesi is one of the provinces that participated in the horticultural village program and the growth of horticultural micro, small and medium enterprises (MSMEs) that receive program assistance in the 2021 fiscal year. The selection is also intended so that horticultural potential outside Java can develop, so that it is expected to reduce dependence from Java, which is the center of Indonesia’s population. In addition, this is expected to be an effort to equalize development programs to grow and develop horticultural centers, especially outside Java. This study used: (i) primary data obtained from program implementers and farmers as program participants.; (ii) secondary data are obtained from the Ministry of Agriculture, Provincial and Regional Agricultural Offices and the Central Bureau of Statistics. Primary data collection was conducted through *Focus Group Discussion*, and direct interviews using structured questions (questionnaires) to program participants. The number of program participants interviewed was 25 chilli farmers in Bantaeng Regency, 10 onion farmers in Bone Regency and 15 potato farmers in Enrekang Regency. Participant consent was written and verbal, where the beneficiary is recorded. Verbally re-conducted when conducting person-to-person interviews.

### Analysis tools

To determine the performance and sustainability of horticultural village activities using context, input, process, product (CIPP) evaluation model. CIPP is needed in compiling research questions whose results are measured using the Rapfish analysis tool (*Rapid Appraisal for Fisheries*). Rapfish is used to evaluate sustainability in a multidisciplinary manner based on ordination techniques (placing things in a measurable order of attributes) with Multi-Dimensional Scalling (MDS [26–28]). MDS itself is basically a statistical technique that attempts to perform multidimensional transformations into lower dimensions. The MDS Rapfish approach is utilized in this research because this tool is popular for evaluating the sustainability of research across a range of disciplines. MDS Rapfish can also be combined with other research techniques to support attribute data and analysis.

The stages of MDS analysis with the Rapfish application include: i) determining attributes, indicators and scoring, ii) determining "good" and "bad" scores then applying them to rapfish, iii) multi-dimensional coordination for each attribute iv) analysis of accuracy and suitability of the model, in this case using value of stress and R-squared (RSQ), v) sustainability level analysis, vi) attribute leverage analysis [29].

Based on the results of the focus group discussion of the research team with other stakeholders (farmers, farmer groups, local government, extension workers, collectors, and researcher), it was agreed that the indicators measured and analyzed in this study include indicators of input, process, product (output) and context (outcome) dimensions. These indicators include elements of sustainability dimensions both from economic, social, institutional, and environmental aspects. In the input dimension of the 18 indicators measured, including: (1) Suitability of the facilitation of the number of seeds, (2) Suitability of seed quality facilitation, (3) Suitability of fertilizer and mulch facilitation, (4) Support for program supporting infrastructure. The four indicators represent relevance to stakeholders and integration of sustainable practices. (5) Availability of capital (economy), (6) Availability of labor for farming activities (social), (7) Availability of corporate/business entities (institutional), (8) Suitability of agroecology (environment).

The thirteen indicators of the process dimensions that were measured, including: (1) Suitability of implementation to the program objectives that have been set, and (2) The level of adoption of agribusiness technology (representing relevance to stakeholders and integration of sustainable practices), (3) Village government support (budget allocation), and (4) Utilization of production facilities (economy), (5) The level of participation of farmer group members in the horticulture village program (social), (6) Intensity of assistance and extension (institutional), (7). Accuracy of program implementation with farmer planting schedule (environment).

The measured product (output) indicators consist of 8 indicators, including: (1) Farmers’ satisfaction with the horticultural village program training (representing relevance to stakeholders, integration of sustainable practices, and social), (2) Increasing the area of horticultural cultivation (economy), (4) The role of corporate/business entities (institutional), (5) Facilitated planting conditions (environment).

Fifteen context (outcome) indicators, including: (1) The rate of increase in horticultural farming income (representing relevance to stakeholders and integration of sustainable practices, (2) The level of selling prices at harvest (economy), (3) Access to marketing of horticultural products (social), (4) The performance of corporate/business entities (institutional), and (5) The increase in productivity (environment).

The MDS approach is a statistical analysis technique that transforms each dimension and multidimensionality of the sustainability dimension of agricultural development programmes [30]. The MDS approach was used in this evaluation, on the basis that it has been shown to produce stable parameter results/approximations [31, 32]. Analyses covered input, process, output and outcome dimensions. Attributes and attribute scoring were determined through FGDs involving experts from various related institutions. The assessment formulation uses variables/indicators for each dimension analysed, namely input, output, process and outcome. Based on this, the consistency of analysis between CIPP and MDS can be combined. The assessment of the level of success and sustainability status of the implemented programme can be concluded on each aspect/dimension analysed.

In MDS, the observed object points are mapped into a two- or three-dimensional space, so that the object points are attempted to be close to the original object. The ordination or distance determination technique in MDS is based on the Euclidean distance squared which in n-dimensional space can be formulated as follows [33].


$$d_{ij}^{2}=\sum {\left(x_{ij}-x_{j}\right)}^{2}$$ (1)

Description: $d_{ij}^{2}$ = euclidean squared distance

*x*_*ij*_ = attribute score value

x_*j*_ = average value of attribute scores

*i* = 1, 2, …, n

*j* = 1, 2, …, p

The ordination of an object point in MDS is approximated by regressing the Euclidean distance (dij) from point i to point j with the origin (dij), which can be formulated through the following equation.


$$\hat{d_{ij}}=\alpha +\beta d_{ij}+e$$ (2)

Description: $\hat{d_{ij}}$ = estimated value

*e* = galat

Typical approximation techniques used to regress the above equations are (1) the least square method and (2) the alternating least squared method based on the root of the Euclidian distance (square distance) or called the ALSCAL method, and (3) the maximum likelihood method. In the context of MDS, the ALSCAL algorithm is the most suitable method to be applied through statistical software/SPSS [34–39].

The ALSCAL method optimises the squared distance to the squared data (origin = dij), which in three dimensions is written in a formula called S-stress as follows [33]:

$$S={\left\{\frac{\left(\sum_{i}\sum_{j}{\left(d_{ij}-\hat{d_{ij}}\right)}^{2}\right)}{\left(\sum_{i}\sum_{j}{\left(d_{ij}\right)}^{2}\right.}\right\}}^{\frac{1}{2}}$$ (3)

Furthermore, in MDS it is necessary to test the goodness of fit, which is nothing but measuring how precisely the configuration of a point can reflect the original data. This goodness of fit in MDS is reflected in the magnitude of the S-stress value. A low stress value indicates a fit model, while a high S-value does the opposite. In Rapfish, a good model fit is indicated by a stress value of less than 0.25 (S < 0.25).

Changes in respondents’ income after participating in horticultural village activities were also measured using a partial budget analysis tool. The partial budget was constructed using a comparison of the adoption of vegetable crop cultivation technology before and after respondents participated in horticultural village activities. In preparation from a partial budget analysis, not all production costs are considered. Costs that vary among systems of management practices are taken into accounted. Variable costs include fertilizer inputs (chemicals and organic fertilizers) and other inputs that support the process of cultivating crops [40, 41]. The partial budget formula used is:

$$Change\;in\;revenue\;\left(profit\;or\;loss\right)=profit\;component–loss\;component$$

$$\begin{array}{l} Change\;in\;revenue\;\left(profit\;or\;loss\right)=profit\;component–loss\;component\\ =\;(added\;income+reduced\;\cos t)-\;(added\;\cos ts+reduced\;income) \end{array}$$

## Results and discussion

### Horticultural village activities and characteristics of respondents

Horticultural village activities are implemented in the form of providing assistance to farmer groups, including superior variety seeds, production facilities, technical guidance activities, and assistance by extension workers. This activity is a transformation of regional activities that are more focused on smaller areas, namely villages. This activity aims to increase farmers’ income, which leads to the achievement of farmers’ welfare.

Respondents who participated in planting vegetables in horticultural village activities had the characteristics: (i) productive age with an average age of 46 years, meaning that farmers participating in the horticultural village programme are at a productive age that is expected to support the performance and sustainability of the programme; (ii) Education for 8 years or graduated from elementary school, to junior high school, meaning that farmers participating in the horticultural village programme are at a level of education that is still relatively low which can be expected to have an influence on the lack of sustainability of the horticultural village programme; (iii) Having experience growing vegetables more than ten years, meaning that farmers participating in the horticultural village programme are at a length of farming experience that is still classified as moderate which is thought to have an influence on the sustainability of the sustainability of the horticultural village programme; (iv) on average cultivated an area of 0.62 ha which indicates that only part of the land (45%) cultivated by farmers has been included in the programme due to the fact that although farmers get incentives in the form of production facilities assistance, but the provision of such assistance is often late from the farmers’ planting schedule; and (v) generally planted with an intercropping system s aimed at reducing farming risks.

### Model suitability

A good model is indicated by an S-stress value <0.25 with an R-square or RSQ value close to 1 [35, 42, 43]. The results of the analysis show that the model formed can be used because it has a stress value of less than 0.25 and the determination coefficient seen at the RSQ value is close to 1 (Table 1) [27, 28, 44]. These results indicate that the attributes of the four dimensions provide accountable and accurate analytical data. Therefore, this model can be used further to see the sustainability of the horticultural village program from the four dimensions and leverage points of sustainability.

**Table 1:** Stress and RSQ value for four dimensions.

Dimension	Stress	RSQ
Input	0.21	0.94
Process	0.24	0.91
Product/Output	0.19	0.96
Context/Outcome	0.21	0.94

Source: primary data processed (author composition)

### Performance of horticultural village activities

#### Input dimensions

The horticultural village programme is oriented towards increasing production through increased productivity and planted area on each vegetable farm. There are three sources of productivity growth in agricultural businesses, namely [45]: (a) technological change towards more advanced technology, (b) increased technical efficiency, and (c) economic scale of business.

Increasing production can be done by using quality production inputs, especially in the used of seeds [46, 47], types and doses of fertilizers [48] and pesticides. The availability of production inputs is important in the horticultural village program. The availability of production inputs provided to each farmer group includes seeds, plastic hood mulch, solid organic fertilizer, liquid organic fertilizer and inorganic fertilizer (Table 1). In addition to increasing production, the input assistance provided is expected to increase farmers’ interest and motivation, increase production, productivity and product quality, and help reduce farming costs.

Production input assistance provided must meet the following conditions: a) Organic fertilizers are registered in Permentan Number 1/Permentan/SR.140/10/2019 concerning organic fertilizer registration; b) Inorganic fertilizers in accordance with Permentan 36/Permentan/SR/10/2017 concerning registration of inorganic fertilizers; c) Biofertilizers registered in Permentan Number 70/Permentan/SR.140/10/2011 concerning biological fertilizers; d) Dolomite registered in Permentan No. 70/Permentan/SR.140/10/2011 concerning soil improvement; e) Mulch registered with the competent authority; f) Plastic (hood for seed seeds) registered with the competent authority.

**Fig 2:**
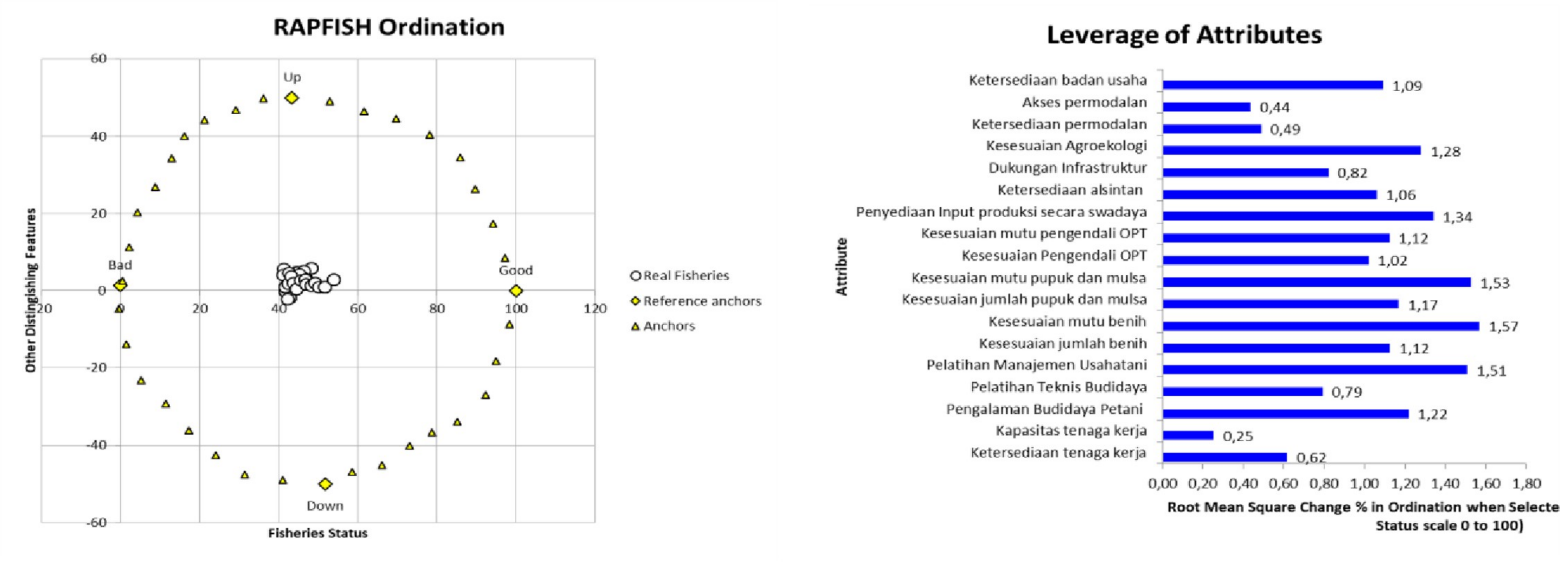
Rapfish graph ordination and leverage of attributes graph of input dimensions. Source: primary data processed (author composition).

The performance position and sustainability status of the horticultural village program from the input dimension are at an index value of 41.86. This indicates that the input dimension is in a less continuous position (Fig 2). There are several sensitivity attributes, which are seen on the rapfish chart, that need to be considered so that horticultural village activities continue to be sustainable. Based on the sensitivity analysis of 18 attributes from the input dimension, three attributes can be seen that are leverage points for the success of horticultural village activities. The three highest attributes began with the suitability of seed quality to the location of the planted land (1.57); suitability of fertilizer quality and type of mulch used (1.53) and farm management training (1.51) (Fig 2).

Seeds are the most important attribute in leveraging the sustainability of horticultural village activities. This is because the seed assistance received by respondents is not in accordance with the quality and variety usually planted by respondents. For chili plants, respondents are accustomed to planting using Rawita or Dewata varieties. While the chili seeds received by respondents were the Baskhara variety. For onion seeds, the quality is not perfect or dormant enough. This condition caused respondents to treat onion seeds for two months, after which the seedlings were ready to be planted. The seeds of assistance obtained by respondents were imported from outside the island of Sulawesi, namely from Java.

#### Process dimensions

In the implementation of the horticultural village program, the area of crops planted was only 45% of the land area owned by respondents. Not the maximum area of land used, because the assistance provided is limited. The assistance is given to farmer groups and later the group administrators will distribute to all their members. The policy of dividing each member is implemented with the aim of equality and can avoid conflicts among members. The lack of maximum land use is seen significantly in potato farmers. Generally, respondents planted potatoes in one hectare using seeds between 1.2 to 1.5 tons. While the number of potato seeds received by respondents was no more than 30 kg.

**Fig 3:**
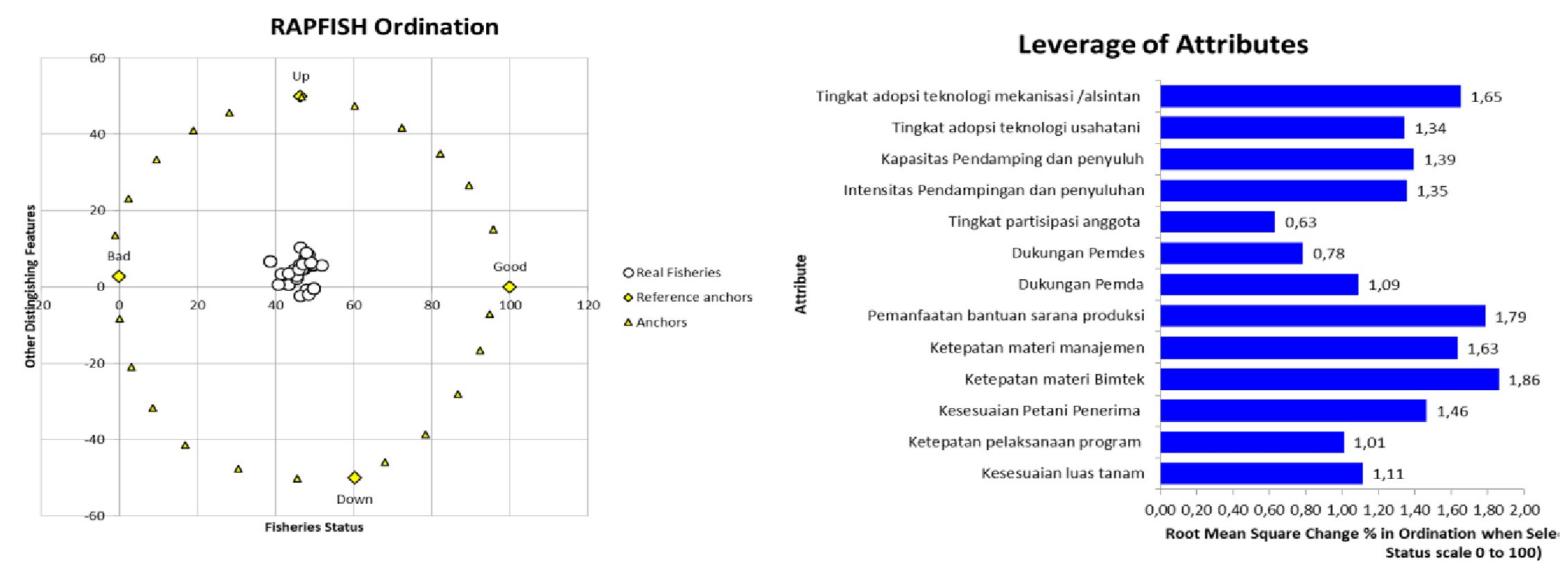
Rapfish graph ordination and leverage of attributes graph of process dimensions. Source: primary data processed (author composition).

The performance position and sustainability status of the process dimension shown in the rapfish graph show an index value of 46.42. This indicates that the process dimension is in a less sustainable position (Fig 3). There are several sensitivity attributes that need to be considered so that the horticultural village program continues. Based on the sensitivity analysis of 13 attributes from the process dimension, especially in the distribution and utilization of aid, it can be seen that three attributes are the leverage points for the success of the horticultural village program. The three highest attributes are seen in: the accuracy of material provision during technology guidance (1.86), the use of production facilities assistance (1.79) and the accuracy of farm management material (1.63) (Fig 3).

#### Product dimension

**Fig 4:**
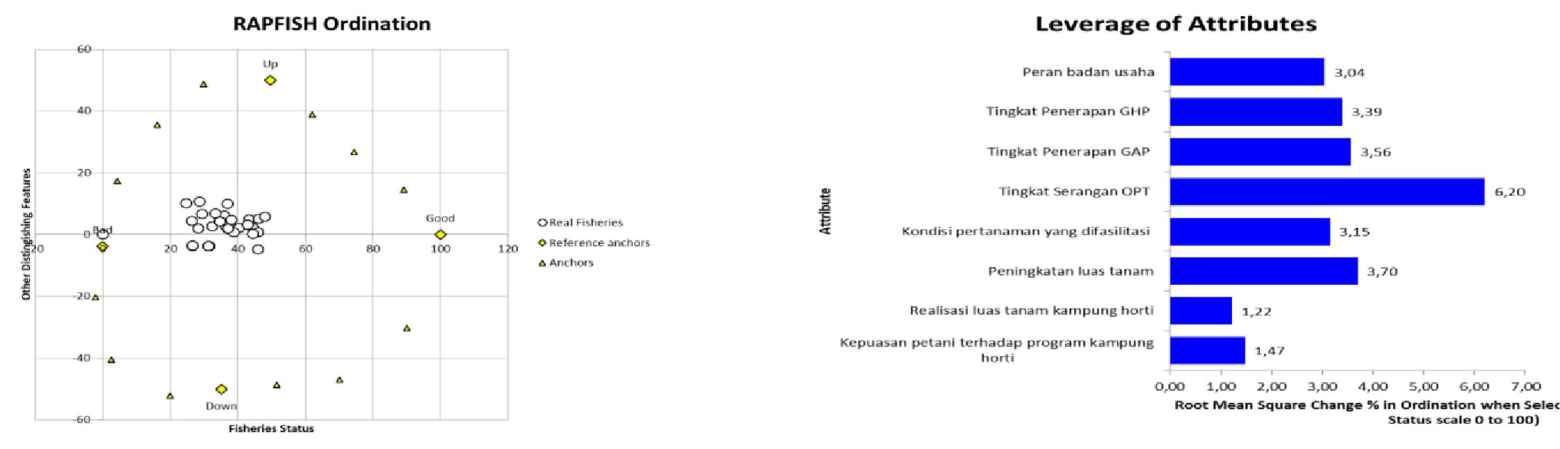
Rapfish graph ordination and leverage of attributes graph of product dimensions. Source: primary data processed (author composition).

The performance position and sustainability status of the product dimension are seen on the rapfish chart with an index value of 34.36. This indicates that the product dimension is in a less sustainable position (Fig 4). There are several sensitivity attributes that need to be considered so that the horticultural village program continues. Based on the sensitivity analysis of 8 attributes from the product dimension, especially in planting conditions, it can be seen that three attributes are the leverage points for the success of the horticultural village program. The three highest attributes were seen: pest control or pest infestation (6.20), increased planting area (3.70) and planting according to GAP (3.56) (Fig 4).

#### Context/outcome dimension

One of the outcomes of this activity is an increase in productivity that can increase farmers’ incomes, thus becoming a catalyst for the sustainability of horticultural crop cultivation and continuity of supply [12]. The increase in productivity of shallots by 1,337 kg / ha, while chili plants experienced an increase in productivity by 1,129 kg / ha. This increase is due to changes in technology adoption from the use of liquid organic fertilizers and NPK Mutiara, which previously did not. On the other hand, by using this type of fertilizer, several other types of fertilizers are no longer used, such as solid organic fertilizer, urea, za and phonska.

**Fig 5:**
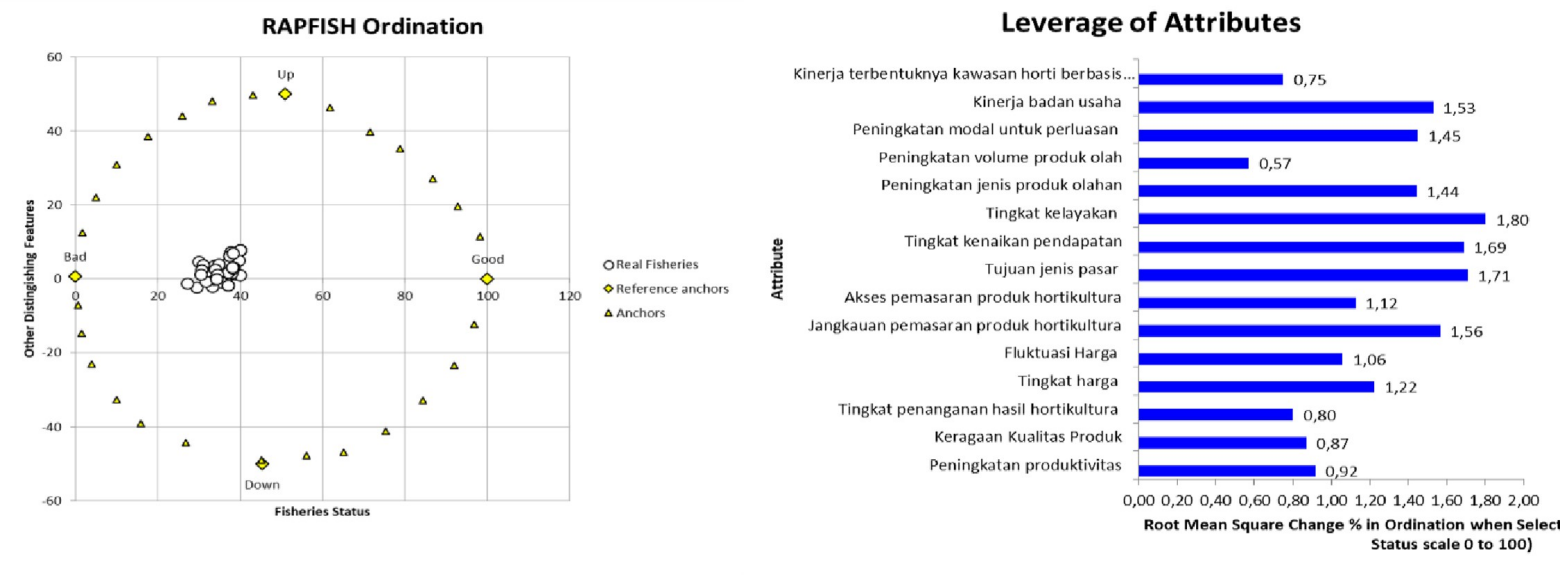
Rapfish graph ordination and leverage of attributes graph of process dimensions. Source: primary data processed (author composition).

On the contrary, potato production actually decreased by 1,830 kg per hectare. This decrease is due to the lack of seed assistance and the mismatch of the size of the assisted seeds with the size of seeds commonly used by farmers. The size of seeds that farmers receive is large, while farmers are accustomed to growing seeds of small sizes. The decrease in potato productivity is also caused when planting coincides with the high rainy season at the site of activity. This condition causes potato plants to be susceptible to pest attacks. From the results of the rapfish graph, it shows that the outcome dimension of this activity is less sustainable, with an index value of 36.18 (Fig 5). There are three levers that need to be considered so that the horticultural village program, especially for the sustainability of vegetable crops. The three factors are: business feasibility level (1.80); there is availability of intended market (1.71) [49] and an increase in revenue (1.69) (Fig 5).

### Changes in respondents’ income

Partial budget analysis is used to determine how much the increase or decrease in production costs is due to the application of new technology. Changes in technology can affect production and production costs. The change was seen in respondents who implemented recommendations for the adoption of vegetable cultivation technology after participating in the horticultural village program (Table 2).

**Table 2:** Types of assistance received by respondents per farmer group per ha.

Commodities	Seeds	Mulch	PO Drop	Solid PO	NPK
Onion	700 kg	5 roll	8 liters	-	150 kg
Chilli	6 sachets	8 roll	7 liters	-	300 kg
Potato	800 kg	-	-	66 kg	150 kg

Source: primary data processed (author composition)

#### Shallots

The results of the study [50] revealed the need for farmers to access advisory services from various sources of information, such as agricultural officers, formal agricultural extension officers, mass media, as well as from the experiences of fellow farmers. It is argued that the key messages from agricultural extension officers differ from farmer-to-farmer messages. Farmer-to-farmer messages focus on encouraging other farmers to work hard and plan and implement measures to finance farming operations, while agricultural extension messages to farmers centre on providing traditional technical tools on group formation for programme assistance, overcoming challenges in marketing, farm diversification and farm management practices.

Changes in cultivation technology that occurred in respondents who planted shallots included: a decrease in the use of solid organic fertilizers, chemical fertilizers of the type of Urea, ZA, Phonska and a decrease in the use of doses of pesticides and fungicides. The decrease in production inputs has led to an increase in the use of liquid organic fertilizers, manure, NPK type chemical fertilizers and soil cover mulch. Additions also occur in onion seeds, because the help seeds received are not optimal.

The reduced use of pesticides and fungicides was caused by respondents following the recommendations of cultivation technology by using plastic mulch. Plastic mulch is defined as the practice of applying a layer of organic, inorganic, or synthetic matter over the soil surface. Some of the benefits of using mulch in cultivation are: maintaining soil moisture and warmth, controlling weeds, improving soil health, reducing topsoil erosion and reduce the use of herbicides.

The reduced use of shallot cultivation technology components has caused farmers to reduce farming costs which are calculated at IDR 4,789,090 per hectare (Table 3). Conversely, by following the recommendations of technology in horticultural village activities, farmers experience an increase in farming costs calculated at IDR 7,099,500 per hectare. Despite the increase in farming costs, overall farmers get an additional income of IDR 8,789,315 per hectare. The cause of the increase in income is due to the production of shallots produced has increased productivity by an average of 637 kg per hectare.

**Table 3:** Analysis of partial budget of horticultural village activities per ha.

Commodities	Addition of technological components				Reduction of technological components			
	Description	Sum	Price (IDR)	Value (IDR)	Description	Sum	Price (IDR)	Value (IDR)
**Shallots**	Seeds	63 kg	23,500	1,480,500	Solid organic fertilizer	50 kg	19,250	962,500
	organic fertilizer drop	8 liters	22,500	180,000	Urea	100 kg	2,500	250,000
	Manure	150 kg	600	90,000	ZA	100 kg	3,000	300,000
	NPK	178 kg	8,000	1,424,000	Phonska	215 kg	6,800	1,462,000
	Mulch	5 roll	785,000	3,925,000	Solid pesticides	6 kg	73,765	442,590
					Liquid pesticides	9 liters	103,000	927,000
					Fungicide	5 liters	89,000	445,000
Sum				7,099,500				4,789,090
Value Difference								(2,310,410)
Production	Increase 637 kg x average selling price per kilogram IDR 17,425							11,099,725
Changes in revenue								8,789,315
**Chilli**	Solid organic fertilizer	4.5 kg	10,000	45,000	Urea	135 kg	2,500	337,500
	organic fertilizer drop	14 liters	41,000	574,000	ZA	123 kg	3,000	369,000
	Manure	25 kg	750	18,750	NPK	120 kg	8,000	960,000
					Phonska	89 kg	6800	605,200
					Solid fungicide	2.5 kg	89,000	222,500
					Herbicides	3 liters	118,000	354,000
Sum				637,750				2,848,200
Value Difference								2,210,450
Production	Increase 349 kg x average selling price per kilogram IDR 21,080							7,356,920
Changes in revenue								9,567,370
**Potato**	Seed	160 kg	10,000	1,600,000	Urea	50 kg	2,500	125,000
	Solid organic fertilizer	112 kg	2,500	280,000	ZA	215 kg	3,000	645,000
	Pesticides	4.5 liters	80,000	360,000	NPK	200 kg	8,000	1,600,000
Sum				2,240,000				2,370,000
Value Difference								130,000
Production	Reduced by 1,120 kg x average selling price per kilogram of IDR 11,500							12,880,000
Changes in revenue								-12,750,000

Source: primary data processed (author composition)

#### Chilli

Changes in cultivation technology in chili plants caused an increase in farming costs which were calculated at IDR 637,750 per hectare (Table 3). This increase is due to respondent farmers having to buy solid organic fertilizer, liquid organic fertilizer and manure. The addition of the use of solid organic fertilizers and manure was previously not a concern for farmers. Even farmers have never tried to use liquid organic fertilizers at all. This is because farmers are accustomed to using chemical fertilizers. As a result of the addition of organic fertilizers, respondents reduced the use of chemical fertilizers of Urea, ZA, NPK and phonska types. In addition to chemical fertilizers, respondents also reduced the use of fungicides and herbicides. Some reductions in the use of production inputs, causing chilli farmers to save farming costs which are calculated at IDR 2,848,200 per hectare. In terms of additional costs, it is estimated at IDR 637,750 per hectare. The increase was due to the fact that respondent farmers had to buy solid organic fertilizers, liquid organic fertilizers and manure. By following the recommendations of horticultural village cultivation technology, chilli farmers experienced an increase in productivity of 349 kg/ha or an increase in income of IDR 9,567,370.

#### Potato

The use of solid organic fertilizers is also recommended for respondents who grow potatoes. In addition to the addition to the purchase of solid organic fertilizers, farmers also added the purchase of pesticides and seedlings. The addition of pesticide purchases is more on the factor of overcoming wet weather due to frequent rain. While the addition of potato seeds is due to the amount of seed assistance is not optimal. The addition of the use of organic fertilizers causes a decrease in the use of Urea, ZA and NPK types of chemical fertilizers.

The adoption of potato cultivation technology by respondents succeeded in reducing the total cost of farming by IDR 130,000 per hectare (Table 3). The addition of farming costs due to the technology adoption that is expected by potato farmers to increase productivity, turns out made an average production decrease by 1,120 kg per hectare. The decrease in production is more caused by rainy season over a long period of time in the planting sites.

## Conclusion

Transformation of the horticultural area approach towards a more focused approach in the horticultural village approach and the growth of Horticultural MSMEs is expected to be more focused programme to perform well and sustainable. Empirically, the performance of the horticultural village programme in the research location was less successful and less sustainable. The lack of sustainability is a pressure point for improvement of the horticultural village programme which is a top-down government programme. The main cause is that the planning and implementation of the programme lacks a mature social process.

The results of the evaluation of the horticultural village development program in South Sulawesi found that the four dimensions, both input, process, product (output), and context (outcome) are included in the category of less sustainable. This shows that serious and focused efforts are needed to continue the horticulture village program to be sustainable. Concrete and gradual steps are needed to give a sustainable impact on horticultural farmers, especially shallots, chillies, and potatoes farmers.

Changes in farmers’ incomes, which are the contex of the program become an entry point to farmers to implemented it, but their evaluation so that the program is sustainable needs to be considered. Improvements can be made in the following aspects: (i) the seeds used are site-specific, because they are in accordance with the local location and farmers are accustomed to planting them (inputs), (ii) the accuracy of extension materials for cultivation technology, and carried out periodically (process), (iii) assistance from extension workers needs to be improved, especially in pest control activities, plant diseases (products), (iv) increasing farmers’ income, especially from increasing productivity or cost reduction from the technology introduced (context).

It is recommended that the implementation of the horticultural village program through the distribution of seeds and inputs be more in line with farmers’ planting schedules and consideration of climatic conditions and market demand. The farming community generally has a planting pattern, planting schedule, and certain superior commodities. Production input assistance that is in accordance or on time with farmers’ planting schedules, according to the needs of farmers and in accordance with the dynamics of market demand and consumer preferences can support success and sustainability.

It is recommended that the provision of technical guidance and farm management and field schools prior to the implementation of the program should be done in a planned manner and through a mature social process, not just a socialisation of the program technical and management guidance includes making organic fertiliser, seed technology, cultivation technology, as well as good harvest handling and processing technology.

Connecting horticulture villages with off takers from the start is necessary to increase the effectiveness of crop maintenance, production and marketing. The problem faced by farmers is not only how to increase the production and quality of good horicultural products, but also whether the production produced is bought at a fair price so that farmers’ income increases. Therefore, the existence of business partnerships that are mutually necessary, strengthening and beneficial can support the sustainability of the program and increase farmers’ income.

This study has limitations because it is only a case study, with a relatively limited number of respondents. Thus, the conclusions obtained do not yet represent Indonesia’s general condition of horticultural agriculture. In addition, the evaluation of the performance and sustainability of horticultural villages is only focused on the dimensions of input, process, product (output) and context (outcome), where the measured indicators have included sustainability elements, namely the relevance of the program to stakeholders, integration of sustainable practices, economic, social, institutional, and environment.

Further studies are needed to see the implementation of Horticultural Villages in other locations in Indonesia. Further studies can use multi-dimensional frameworks such as the Triple Bottom Line (TBL) or Sustainable Development Goals (SDGs) so that they are more comprehensive. It is also suggested to add references related to the application of frameworks, methods, or tools on the sustainability of development programs. In addition, alternative studies can also be carried out by expanding the scope of aspects such as: entrepreneurship index, commercialization index, and digitization marketing.

## Supporting information

S1 Data (XLS)
